# Microglial P2Y_12_ Deficiency/Inhibition Protects against Brain Ischemia

**DOI:** 10.1371/journal.pone.0070927

**Published:** 2013-08-05

**Authors:** Corey M. Webster, Masaaki Hokari, April McManus, Xian Nan Tang, Hualong Ma, Rachid Kacimi, Midori A. Yenari

**Affiliations:** Department of Neurology, University of California, San Francisco and the San Francisco Veteran’s Affairs Medical Center, San Francisco, California, United States of America; Virginia Commonwealth University, United States of America

## Abstract

**Objective:**

Microglia are among the first immune cells to respond to ischemic insults. Triggering of this inflammatory response may involve the microglial purinergic GPCR, P2Y_12_, activation via extracellular release of nucleotides from injured cells. It is also the inhibitory target of the widely used antiplatelet drug, clopidogrel. Thus, inhibiting this GPCR in microglia should inhibit microglial mediated neurotoxicity following ischemic brain injury.

**Methods:**

Experimental cerebral ischemia was induced, *in vitro* with oxygen-glucose deprivation (OGD), or *in vivo* via bilateral common carotid artery occlusion (BCCAO). Genetic knock-down *in vitro* via siRNA, or *in vivo* P2Y_12_ transgenic mice (P2Y_12_−/− or P2Y_12_+/−), or *in vivo* treatment with clopidogrel, were used to manipulate the receptor. Neuron death, microglial activation, and microglial migration were assessed.

**Results:**

The addition of microglia to neuron-astrocyte cultures increases neurotoxicity following OGD, which is mitigated by microglial P2Y_12_ deficiency (P<0.05). Wildtype microglia form clusters around these neurons following injury, which is also prevented in P2Y_12_ deficient microglia (P<0.01). P2Y_12_ knock-out microglia migrated less than WT controls in response to OGD-conditioned neuronal supernatant. P2Y_12_ (+/−) or clopidogrel treated mice subjected to global cerebral ischemia suffered less neuronal injury (P<0.01, P<0.001) compared to wild-type littermates or placebo treated controls. There were also fewer microglia surrounding areas of injury, and less activation of the pro-inflammatory transcription factor, nuclear factor Kappa B (NFkB).

**Interpretation:**

P2Y_12_ participates in ischemia related inflammation by mediating microglial migration and potentiation of neurotoxicity. These data also suggest an additional anti-inflammatory, neuroprotective benefit of clopidogrel.

## Introduction

Microglia have received much attention recently in terms of their contribution to a variety of acute neurological injuries. There are many known receptors and pathways that have been shown to activate the microglial inflammatory cascade. The family of purinergic receptors have gained interest because their ligands include nucleotides that may be released by injured cells, which bind these receptors and activate a diversity of activities involved in neurodegeneration and ischemic cell death, including proinflammatory signaling, chemotaxis, and phagocytosis [1].

The role of purinergic receptors in the microglial inflammatory response has largely focused on P2X_7_, where it has been shown to modulate microglial activation following experimental brain ischemia and stroke, and its pharmacologic blockade led to decreased ischemic damage [2], [3], [4], [5]. However, little has been studied on the Gi coupled ATP receptor, P2Y_12_ [1], [6]. P2Y_12_ is present on microglia, and is expressed on the microglial membrane surface in the resting state and activated by ATP, ADP, or neighboring neurotoxicity [7], [8]. It is internalized when microglia are activated [7], [9] and promotes microglial migration toward the source of these nucleotides [7], [10]. Furthermore, it has been shown that P2Y_12_ may also be involved in the microglial inflammatory cascade as ADP induces phosphorylation of Akt [11]. While P2Y_12_ has been shown to modulate microglial migration, it has also been shown not to be involved in controlling microglial phagocytosis, which is the role of a different G protein coupled purinergic receptor, P2Y_6_ [12]; hence the purinergic receptors for ADP vs UDP act in a mutually exclusive manner.

Because P2Y_12_ is also the target of a widely used antiplatelet agent, clopidogrel, it is a very attractive target for assessing its role in the microglial mediated inflammatory cascade. Here we evaluate the role of P2Y_12_ in ischemia pathogenesis as it pertains to microglial activation.

## Materials and Methods

All animal experiments were approved by the Institutional Animal Care and Use Committee of the San Francisco Veterans Affairs Medical Center (IACUC, protocol numbers 10-067 & 10-074) and were in accordance with NIH guidelines. All experiments were randomized, and data were analyzed in a blinded manner.

### Cell Cultures

Primary neuron, astrocyte and microglial cultures were prepared using previously established methods from our lab [13], [14]. Astrocytes were prepared from C57/BL6 P1-3 mouse pups. When astrocytes were 14 DIV, primary neurons were prepared from ∼E15 C57/BL6 mouse embryos and plated on top of astrocytes. When neurons were 8 DIV, primary microglia were harvested from astrocyte cultures by the shake off method [15] and plated on top of the neuron-astrocyte cultures at a density of 1–3×10^4^ cells/ml or at an approximate ratio of 1∶10:10 microglia :neuron: astrocyte, and allowed to stabilize for 24 h. In other experiments, BV2 cells (a murine microglial cell line) were plated on top of the neuron-astrocyte cultures at a similar density and ratio, or placed in inserts (pore size = 0.4 µm) above the neuron-astrocyte cultures. Co- and tri-cultures of neurons, astrocytes with or without microglia (or BV2 cells) were prepared, or plated in inserts (pore size 0.4 µm) above neuron/astrocyte cocultures. All cultures were maintained in 5% CO_2_.

### Oxygen Glucose Deprivation (OGD)

To simulate ischemic conditions, cultures were exposed to OGD as previously described [13]. Cultures were maintained in an anoxia chamber for 1 h at 37°C, unless otherwise specified, and oxygen tension was maintained at <0.001% (Coy Laboratories). Media was removed and cultures were washed three times with balanced salt solution (BSS0) lacking serum or glucose, or oxygen. Control cultures were incubated at normoxia with balanced salt solution containing 5.5 mM glucose (BSS5.5). After 1 h of OGD, glucose was added to each well to a final concentration of 5.5 mM, and plates were incubated at normoxia in a regular incubator for 23 h at 5% CO_2_, at 37°C.

### Neuron Viability Assay

Neuron viability assay methods were conducted in accordance with previously described methods, based on the detection of MAP-2 by ELISA (Brooke et al. 1999). Culture wells were fixed with 50% ethanol: 50% acetone for 15 min at −20°C, then washed three times with PBS. The wells were blocked with 5% non-fat dried milk in PBS (5% NFDM) for 1 h, washed, and anti-MAP-2 mouse antibody (clone HM-2, Sigma, St. Louis, MO; 1∶1000 dilution) was added in 5% NFDM to each well and incubated at room temperature for 30 min. The wells were washed again, and biotinylated anti-mouse antibody (Vector Laboratories, Burlingame, CA; 1∶200 dilution) in 5% NFDM was added and incubated at room temp for 30 min. After washing, 50 µl of ABC reagent (Vector Laboratories, Burlingame, CA) was added to each well for 30 min at room temp. Wells were washed again four times with PBS, then 50 µl of ABTS (Vector Laboratories, Burlingame, CA) reagent was added and developed for 5–20 min while light-shielded. The reaction was stopped using 50 µl of 1% SDS in water, and 50 µl of sample from each well was then transferred to another 96 well plate for reading in a spectrophotometric platereader (Beckman Coulter DTX880) at 405 nm.

### Cluster Counts

The clustering of microglia observed in mixed cultures was quantified using the following method. “Cluster” was defined as a group of microglial cells, appearing adherent to each other rather than evenly distributed, as they appeared upon plating. Clusters were counted and scored based on the size of the cluster: 0–9 cells in a cluster, a score = 1, for 10–99 cells = 2, 100–999 cells = 3. This score was multiplied by the number of clusters in each field, and a “cluster score” was computed. At least five non-overlapping fields were counted for each experimental condition.

### Microglial Migration Assay

Chemotaxis was assessed in two ways. The first method estimated the ability of cells to move through a cell culture insert using a CytoSelect 5 µm pore, 96 well transwell cell migration assay (Cell Biolabs, CBA-105, pore size 5 µm) according to the manufacturers’ protocol. Supernatants from neurons treated with OGD for 2 h followed by 1 h repurfusion were used to test chemotaxis. We selected this OGD paradigm on the neurons as well as neurons plus astrocytes for optimal ATP or ATP hydrolyzed product secretion into the media. BV2 cells were plated at a density of 1×10^5^ cells/ml in the membrane chamber in serum free media. Wells of the feeder tray contained either control or experimental media. BSS5.5 was used as control media. Experimental media consisted of supernatants from neurons or mixed cultures that had undergone OGD as described above, with cells ‘reperfused’ in BSS5.5 by adding glucose to the media. The assay tray was incubated in a 5% CO_2_ incubator at 37°C for 2 h. The cell membrane tray was separated from the lower feeder tray, and any adherent cells were dislodged using solutions provided in the kit, and transferred to a new 96 well plate. Cell lysis buffer and CyQuant GR solution was added and incubated for 20 min, then 150 µl aliquots were carefully transferred to a new 96 well plate for fluorescence detection using a fluorescent plate reader at 480 nm/520 nm (Beckman Coulter DTX880). Each experiment was carried out in 6 wells and was repeated a minimum of three times.

Migration was also studied using a Dunn chamber with OGD supernatants from OGD injured neurons, enriched neurons, or a no OGD control were used as a chemotactic stimulus in a manner similar to that previously described [7]. Primary microglia or BV2 cells were plated at a density of 1×10^5^ cells/ml onto glass coverslips and allowed to settle in an incubator for 48 h after plating prior to use in experiments. Coverslips were washed 3x with BSS5.0 solution, then inverted and secured onto a Dunn chamber (Hawksley, # DCC100, UK) using a mixture of 50% petroleum gel (Vasoline) and 50% melted paraffin wax to avoid evaporation of media. Upon placement of the coverslip, both the inner and outer wells of the Dunn chamber contained control media (BSS5.0). The outer well was then changed to either control or experimental media consisting of supernatant from neurons and/or astrocytes from the above OGD studies. Cultures exposed to OGD were centrifuged to eliminate cell debris in the supernatants. Cells were then time lapse imaged at a rate of 1 image per 5 minutes on an Axiovert inverted fluorescence microscope, 20X objective (Carl Zeiss) for 1 h. Ambient temperature was maintained at 37°C during imaging. The overall distance traveled by individual cells over a 30 min period was determined by marking the center of each cell, and determining its coordinates in an x-y plane, and distance was computed by determining the length between coordinates.

### Gene Knockdown of Microglial Cells

The immortalized microglial cell line, BV2 were cultured in RPMI media (UCSF Cell Culture Facility, San Francisco, CA), supplemented with 10% FBS Defined (Hyclone, Waltham, MA) and penicillin/streptomycin. BV2 cells were transfected with P2Y_12_ or control siRNA, optimized for reverse transfection and optimal knockdown with minimal BV2 cell activation and death. In serum free OptiMEM media (UCSF Cell Culture Facility, San Francisco, CA), Lipofectamine (Invitrogen, Carlsbad, CA) reagent was incubated for 15 min. at a concentration of 3.6 ul reagent to 1.5 ml OptiMEM. Concurrently, PLUS reagent (Invitrogen, Carlsbad, CA) 15 ul per 1.5 ml was mixed with the siRNA at a concentration of 10.8 ul siRNA (Ambion, Foster City, CA) (from a stock of 50 uM)/1.5 ml OptiMem or 5.4 ul siRNA, when two different targets of the same gene were used. The final concentration of siRNA was 30 nM. The 1.5 ml OptiMEM containing Lipofectamine was mixed and incubated for 30 min with the equivalent volume of OptiMEM containing the siRNAs and PLUS reagent. BV2 cells were then washed with PBS, trypsinized (0.25% Trypsin, UCSF Cell Culture Facility, San Francisco, CA), and plated at 1.25×10^5^ cells in wells of a 6 well plate containing 0.5 ml of the mixture of transfection reagent and siRNAs for a reverse transfection. Cells were allowed to freely float in reagent until adhering to the bottom of the plate, rather than transfecting them after they were adherent. OptiMEM, 0.5 ml, was added to each well so that the final concentration of siRNA was 30 nM. The BV2 cells were allowed to incubate in the reagent for 4 h at 37°C in 5% CO_2_, then the media was exchanged with antibiotic free RPMI plus 10% FBS.

BV2 cells were allowed to rest for 48 h after transfection prior to plating onto primary neuron-astrocyte cultures.

### Global Cerebral Ischemia Model

8–12 week old male mice were used. P2Y_12_ knockout mice (C57/BL6 background) and wildtype littermates (WT) obtained from David Julius and colleagues [7] were used. P2Y_12_-heterozygous mice (P2Y_12_+/−, n = 11) and wildtype littermates (WT, n = 7) and WT littermates treated with clopidgrel (CL, n = 6 were compared). Among treated mice, clopidogrel was given in a dose of 5 mg/ml PBS, with 5 ml/kg po (25 mg/kg) given as gavage feeds. Clopidogrel was given 2 h prior to ischemia, followed by repeated doses at 1 and 2 days post ischemia. This dose was based on a prior study that showed that clopidogrel’s effects could be seen in the nervous system after oral dosing [16]. Pilot studies of GCI from homozygote P2Y_12_^−/−^ knockout mice revealed that these mice suffered higher mortality than the wild type GCI mice, and the extent of neuron damage was highly variable. Thus, it was not possible to collect useable data, and only +/− heterozygote knockout mice could be used. This could be due to indirect effects of changes in homeostasis resulting from complete P2Y_12_ deficiency among the homozygote knockouts. Homozygote P2Y_12_ knockout mice have also been documented to possess altered platelet function as evidenced by prolonged bleeding times [17].

Global cerebral ischemia (GCI) was induced for 12 min via bilateral common carotid artery occlusion (BCCAO) under isoflurane anesthesia [18]. Core temperature was recorded and maintained between 36.5–37.5°C during the procedures. Animals were assigned to randomly treatment groups, or different genotypes were studied in a random fashion to avoid bias.

72 h later, the animals were euthanized via isoflurane overdose and transcardially perfused with normal saline, followed by 5 ml of 2.5% carbon black in order to assess the posterior communicating artery (PComm) [19]. After brains were removed, the plasticity of the each PComm was graded on a qualitative scale of 0 to 2 with 0 = no visible PComms, 1 = only one PComm or PComm’s with a diameter of less than one third of that of basilar artery, 2 = PComms with a diameter of more than one third of that of basilar artery. As a result, the plasticity of each mice of PcomA was graded on a qualitative scale of 0 to 4. After harvest, brains were sunk in 20% sucrose, and frozen at −80°C stored until use. Frozen sections were prepared in the coronal plane (20-µm-thick) using a cryostat.

To confirm whether there were differences between genotypes in cerebral blood flow (CBF) reduction during BCCAO or not, CBFs of a separate set of 4 mice (2 P2Y_12_+/− and 2 WT mice) were studied. CBF was studied using a laser-Doppler flowmetry (OXYLAB LIF, OXFORD OPTRONX). Two 1.5 mm holes were drilled 3 mm lateral and 1 mm caudal from bregma, and the dura was opened carefully. The laser Doppler probe was secured perpendicular to the brain surface. Ten readings (2 seconds apart) of local CBF (lCBF) were collected from the digital display of the LDF unit and were averaged to provide an lCBF value. The initial CBF reading was recorded as 100% and subsequent flow changes (5 and 10 min after BCCAO and 5 min after reperfusion) were expressed relative to this value. After CBF measurements were taken, these 4 mice were euthanized then assessed for PComm plasticity as described above.

### Western Blots

Whole cell lysates were harvested from cells or brain tissue, and protein concentration was determined by a Bradford Protein assay. Lysates were run on a 10% Tris gel (Bio-Rad, Hercules, CA), and transferred to nitrocellulose membranes. The membranes were probed with an antibody against P2Y_12_ (1∶250 dilution, #APR-012, Alomond Labs), followed by secondary antibody then reacted with ECL.

### Histochemistry and Fluorescent Microscopy

To estimate neuronal death in the hippocampal CA1 sectors, coronal sections 72 hr post 12-min global cerebral ischemia were stained with hematoxylin-eosin (H&E). The numbers of morphologically ischemic and viable neurons were counted in blinded fashion from the CA1 pyramidal cell layer by sampling 10 non-overlapping fields at high power [18].

Microglia were identified from IB4 stains (Han et al. 2002). Brain sections were treated for endogenous peroxidases with 0.03% hydrogen peroxidase. Sections were then incubated in peroxidase-labeled Griffonia simplicifolia isolectin-B4 (IB4) (10 ug/ml, GSAI-B4; Sigma L5391) overnight at 4°C, followed by diaminobenzidine (Vector laboratories, Burlingame, CA, USA) [20].

Immunostains of nuclear factor kappaB’s (NFkB) p56 subunit (1∶50, sc-109, Santa Cruz) were carried out on adjacent sections. All p65 immunoreactive cells in hippocampal CA1 from coronal sections were counted, as well as cells with nuclear staining. After hydrogen peroxidase treatment, brain sections were blocked with 5% BSA, and reacted with antibodies against p65 (1∶50, sc-109, Santa Cruz) overnight at 4C, followed by a biotinylated secondary antibody. The secondary antibody was detected using the Elite Vectastain ABC kit (Vector laboratories) and diaminobenzidine, then counterstained with hematoxylin.

In all cases, brain tissue incubated without the primary antibody or substrate but processed with all secondary and tertiary regents served as the negative control.IB4 positive microglia were identified by the deeply brown-stained cells in the CA1 region and the region between the blades of the dentate of high power fields from coronal hippocampal sections. All p65 immunoreactive cells in hippocampal CA1 from coronal sections were counted, as well as cells with nuclear staining.

In order to identify NFκB positive microglia, double immunofluorescent labeling was carried out for p65 and CD11b. Brain sections were blocked, incubated in antibodies against p65, followed by Cy3-conjugated goat anti-rabbit IgG (H+L) (1∶200, 56,339; Jackson Immuno Research, West Grove, PA,USA) at room temperature for 1 h. The sections were washed, then reacted with FITC-conjugated anti-mouse CD11b (1∶200, Becton Dickenson) at room temperature for 3 h. Sections were then mounted on glass slides using Vector-Shield mounting medium. Sections were then viewed on a Zeiss Aviovert 40 CFL fluorescent microscope (Germany).

### Statistics

All data analysis was carried out by investigators blinded to experimental conditions. Standard statistical tests were applied depending on the condition. ANOVA or T-test were applied for continuous (normally distributed) data, such as neuron viability, cell counts, relative fluorescence and distance traveled (migration assays). Non-parametric tests (Wilcoxon Rank sum) were applied for non-continuous data such as mortality, PComm and cluster scores) and analyzed using SigmaStat. Data are presented mean ± SE unless otherwise noted.

## Results

### Microglia Increase Neuron Susceptibility to Ischemia-like Insults

**Figure 1:**
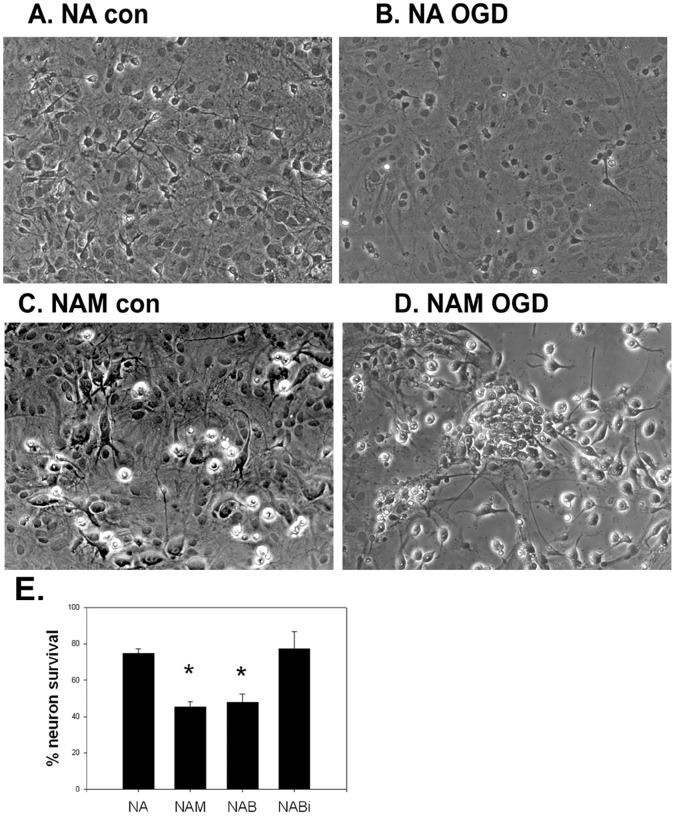
Microglia potentiate neuron death following in vitro ischemia. Representative images of mixed neuron astrocyte (NA) cultures prior to oxygen glucose deprivation (OGD) (A, NA con) and after (B, NA OGD). Primary microglial cells added to NA cultures are visible as small phase bright cells (C. NAM con). Following OGD, the previously confluent NA cell layer is disrupted leaving mostly microglial cells which tend to form clusters (D, NAM OGD). E: Mixed cultures of primary neurons and astrocytes (NA) were prepared and either primary microglia (NAM) or BV2 cells were added (NAB). Neuron death due to OGD was increased in mixed cultures containing either primary microglia (NAM) or BV2 cells (NAB), and the extent of death was similar. Microglia appear to require cell-cell contact, since BV2 cells did not potentiate neurotoxicity when cultured in inserts (NABi). (*P<0.001 vs. NA, NABi).

We previously established that primary microglia increased primary neuron cell death due to OGD [21]. These findings were replicated here whether we used primary microglia or BV2 cells. The addition of microglia to neuron-astrocyte cultures halved the amount of neuron survival (Fig. 1). We then determined whether cell-cell contact is required for this neurotoxicity by culturing microglia in inserts suspended above a monolayer of neurons and astrocytes, but cell viability was not affected by this manipulation. These data indicate that microglia require direct contact with neurons and/or astrocytes to elicit neuron cell death.

### P2Y_12_ Deficiency is Protective

**Figure 2:**
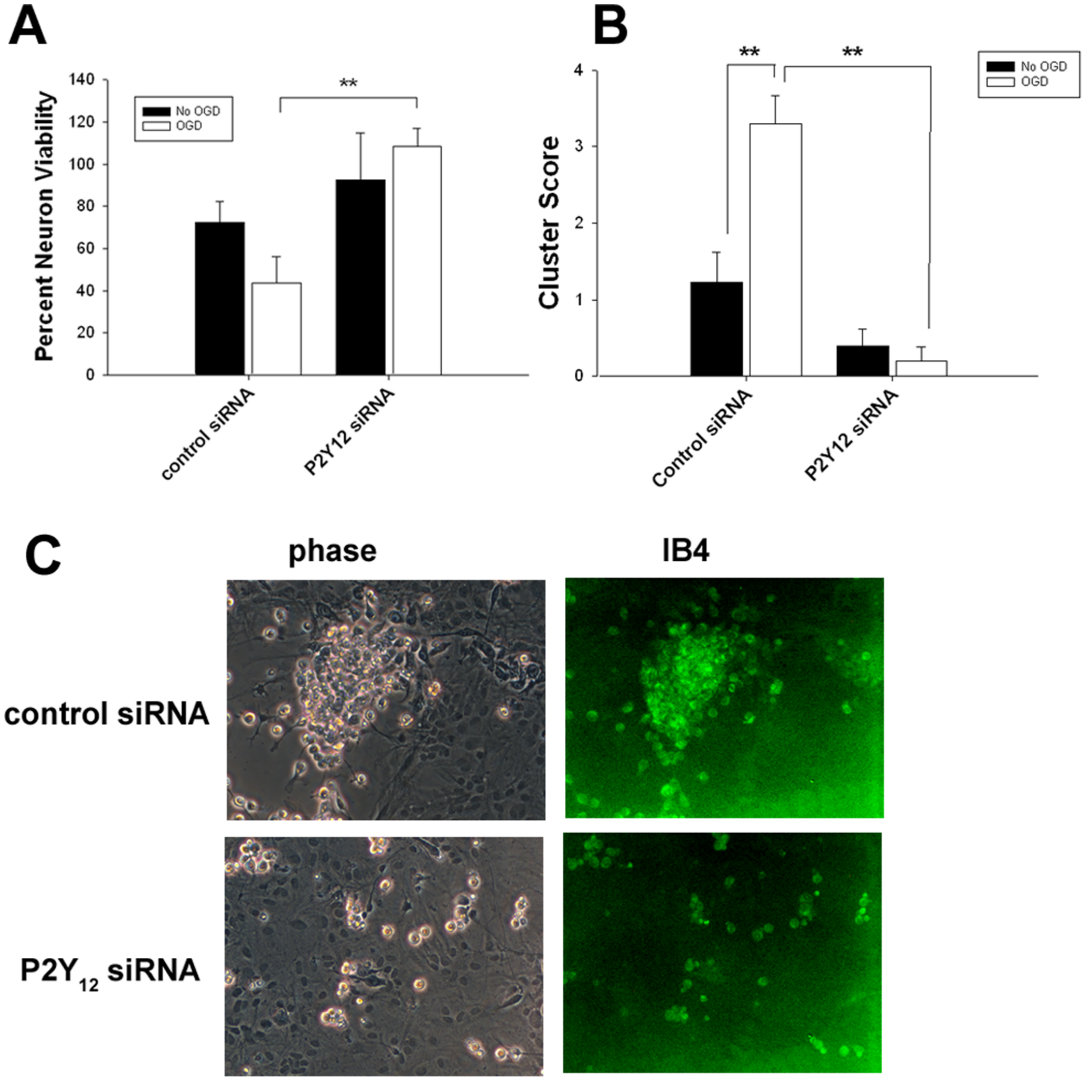
P2Y_12_ deficiency in microglia improved neuron survival following OGD and prevented microglial clustering. P2Y_12_ was knocked down in BV2 cells using siRNA, and were cocultured with mixed neuron-astrocyte cultures. Scrambled siRNA was used as a control. Following OGD, neuron viability was decreased in BV2 cells receiving control siRNA, but was increased where P2Y_12_ was deficient (A). The tendency towards microglial clustering was increased by OGD, but prevented by P2Y_12_ deficiency (B, C). Immunostains of the microglial marker isolectin B4 (IB4) indicate that the majority of the cells forming the clusters are indeed microglia (C). **P<0.01.

To determine whether microglia-potentiated neurotoxicity following OGD was due to P2Y_12_, P2Y_12_ was knocked down in BV2 cells using siRNA (Fig. S1), then co-cultured with neuron-astrocyte cultures. Mixed cultures were exposed to OGD for 1 h followed by 23 h reperfusion. Neuron viability was improved in P2Y_12_ deficient BV2 cells (Fig. 2A). We also noticed that microglia tended to form clusters around dying cells following OGD, confirmed by isolectin B4 (IB4) staining (Fig. 2C). Clustering was significantly decreased in P2Y_12_ deficient BV2 cells (Fig. 2B–C).

### Microglia Migration Studies

**Figure 3:**
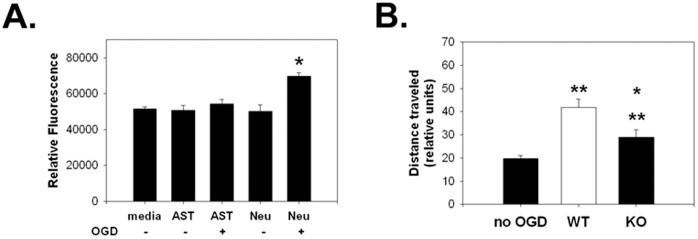
Microglia migrate in response to OGD injured neurons, but not astrocytes. Migration is inhibited under conditions of P2Y_12_ deficiency. A: A Boyden-like assay was used to estimate the ability of BV2 cells to travel through a cell culture insert. BV2 cells were placed in inserts, which were then placed above wells containing various potential chemotactic stimulants. Cells migrating into the lower chamber were then quantified using a fluorescent detection dye. Test media are indicated in the upper row of the x-axis label (media = BSS 5.5; AST = astrocyte-conditioned media; Neu = neuron-conditioned media). Cultures previously exposed to OGD are indicated by ‘+’, and those not exposed by ‘−’. OGD increased BV2 cell migration neuron conditioned media, but not astrocyte conditioned media. B: Using a Dunn chamber assay where distance traveled can be estimated, primary microglia harvested from wildtype (WT) or P2Y_12_ deficient (P2Y_12_^−/−^, KO) mice, and were exposed to conditioned media from OGD-exposed wildtype neurons. The distance traveled over a 30 minute observation period is shown. Primary microglia from KO mice traveled shorter distances than WT microglia. (*P<0.05, **P<0.01).

Since P2Y_12_ is involved in chemotaxis, we determined whether neurons or astrocytes exposed to OGD release migratory signals into their respective supernatants. Primary astrocytes or primary neurons were exposed to 2 h OGD followed by 1 h reperfusion, a protocol designed for optimal OGD induced release of ATP or hydrolyzed products into the supernatant [22], [23], and supernatants were collected for migration studies. Interestingly, BV2 cells consistently migrated toward OGD-conditioned neuron supernatants, but not toward uninjured control neurons and not toward either the OGD-exposed or uninjured control astrocyte supernatants (Fig. 3A). Thus, microglia appear to be more inclined to migrate toward injured neurons versus uninjured neurons and injured and uninjured astrocytes via secreted factors.

We then prepared primary microglial cultures isolated from wildtype or knockout P2Y_12_ (−/−) mice and determined their motility towards OGD-conditioned neuron supernatants using a Dunn chamber assay. In response to OGD-exposed neuron supernatants, wildtype microglia traveled farther than P2Y_12_ deficient microglia and wildtype microglia exposed to supernatants from uninjured neurons (Fig. 3B).

### P2Y_12_ Deficiency or Inhibition by Clopidogrel Improved Hippocampal CA1 Neuron Viability and Decreased Immune Responses Following BCCAO

Physiological parameters were no different between experimental groups, and PComm scores and CBF were no different between WT and P2Y_12_ KO mice (Fig. S1b, Table 1–2). Mortality was also not affected by genotype or treatment (Table 1).

**Table 1:** Mortality & PComm scores in P2Y_12_ deficient & Wildtype mice.

Group	Mortality	PComm Score
P2Y_12_+/−	0/6	0.3±0.2
WT	2/11	0.2±0.4
WT+clopidogrel	0/5	NT

**Table 2:** CBF during BCCAO by LDF.

	5 min pre	5 min post	10 min post	5 min into reperfusion
WT (%CBF) mean±SD, n = 4	100±0	16.4±4.2	12.4±2.9	98.3±8.4
P2Y_12_^+/−^ (%CBF) mean±SD, n = 4	100±0	15.0±4.5	10.4±2.1	102.6±7.2

**Figure 4:**
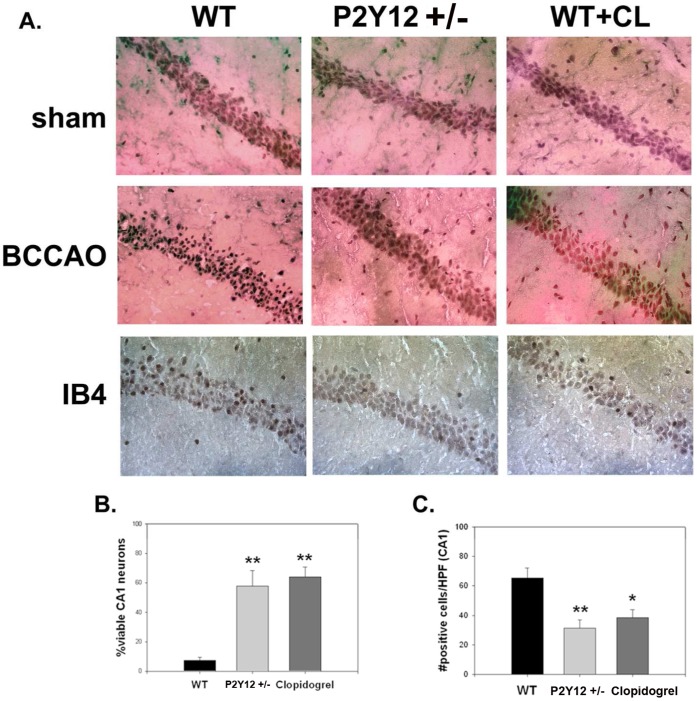
Mice deficient in P2Y_12_ are protected from global cerebral ischemia and also have decreased microglia surrounding areas of injury. Following bilateral common carotid artery occlusion for 12 min followed by 3 d reperfusion, P2Y_12_ deficient mice (P2Y_12_^+/−^) suffered less injury to hippocampal CA1 compared to wildtype (WT) mice. WT mice treated with clopidogrel (WT+CL, Clopidogrel) were also protected (A, B). Following BCCAO, microglial densities as estimated from counts of isolectin B4 (IB4) positive cells were also decreased within CA1 among P2Y_12_^+/−^ and Clopidogrel treated mice (A, C). *P<0.05, **P<0.01.

The percentage of viable neurons in hippocampal CA1 was significantly higher in P2Y_12_+/− and clopidogrel treated mice than in WT mice (Fig. 4A–B). Clopidogrel treatment also increased the percentage of viable CA1 neurons (CL35.9±19.9% cell death, P<0.001). BCCAO also increased densities of microglia, as identified by IB4 staining around hippocampal CA1 and between the blades of the dentate, both of which were significantly decreased by P2Y_12_ deficiency and inhibition by clopidogrel (Fig. 4A, C).

**Figure 5:**
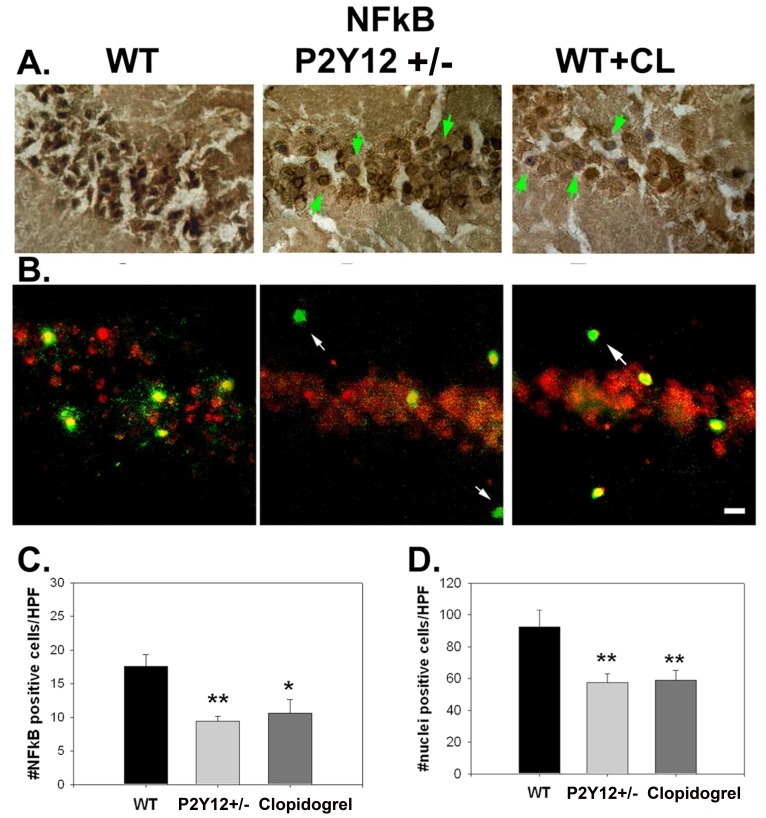
P2Y_12_ deficiency or inhibition decreases NFκB expression. Numbers of cells positive for NF**κ**B’s p65 subunit were decreased among P2Y_12_^+/−^ and treated mice (A, C). Furthermore, cells of P2Y_12_^+/−^ and treated mice had less nuclear NF**κ**B staining (A, green arrows) compared to untreated WT following BCCAO (A, D). B: Double labeling for NF**κ**B’s p65 subunit (red) and the microglial marker CD11b (green) show that most CD11b cells are also positive for NF**κ**B after BCCAO in WT brains. Several microglia in brains of P2Y_12_^+/−^ mice or WT mice treated with clopidogrel (WT+CL) are not NF**κ**B positive (arrows). *P<0.05, **P<0.01, scale bar = 25 µm.

BCCAO increased nuclear translocation of the pro-inflammatory transcription factor, NFκB. Many cells within CA1 were darkly stained for NFκB’s p65 subunit; however, less nuclear staining was observed in similar brain regions in P2Y_12_ deficiency or clopidogrel treatment (Fig. 5A, C–D). Co-labeling with a microglial marker also showed decreased p65 in these cells of knockout or clopidogrel treated mice (Fig. 5B).

## Discussion

We show here that microglia potentiate acute injury in the setting of brain ischemia and ischemia-like insults. This toxicity appears to be mediated the P2Y_12_ purinergic receptor, which controls microglial chemotaxis. Deficiency or blockade of this receptor leads to neuroprotection. P2Y_12_ is also the receptor against which the antiplatelet agent, clopidogrel acts and suggests an additional therapeutic benefit of this commonly prescribed drug.

Microglia have been viewed as both neuroprotective or neurotoxic in the context of cerebral ischemia and other neurological diseases [24]. A substantial body of literature appears to support the notion that, at least acutely, microglia contribute to the evolution of brain cell death following ischemia [25], [26]. Yet, others have shown that microglia protect the brain from injury. In a model of neonatal brain ischemia, depletion of brain resident microglia increased levels of inflammatory cytokines and exacerbated the focal injury 72 h after injury onset [27]. Studies employing media transfer also showed that microglia-conditioned media protected neurons from excitotoxicity and apoptosis [28], [29]. However, in our models, we previously showed that microglia increase ischemia-like injury to neurons [21], and that the protective effect of mild hypothermia was associated with the suppression of multiple pro-inflammatory pathways in microglia [18], [20], [30]. Here we further demonstrate that microglia require cell-cell contact for neurotoxicity following OGD, thereby requiring the migration of microglia to target cells.

The microglial purinergic receptor, P2Y_12_, mediates neurotoxicity. Purinergic receptors are found in numerous cell types, both in brain and peripherally [31], [32], and mediate a variety of cellular functions. The P2 purinoreceptors consist of two families: the ionotropic receptors (P2X) contain channels that permit ion flow, whereas the metabotropic receptors (P2Y) are G-protein coupled second messenger systems [6], [33]. In immune cells, they are involved in pro-inflammatory responses [1], migration [7] and phagocytosis [12].

Purinergic systems have not been extensively studied in brain injury models. A few reports have focused on the ionotropic P2X_7_ receptor, and its inhibition or blockade has been shown to decrease microglial responses and improve neurological outcome from experimental stroke and global cerebral ischemia [2], [4], [5]. Treating rodents with a non-selective P2 blocker, Reactive Blue 2 improved outcome from experimental stroke with differential expression of P2X_7_ [3]. However, less has been studied of the P2Y_12_ receptor in brain injury models. To our knowledge, this is the first report of a potential neuroprotective role of P2Y_12_ in brain ischemia.

The P2Y_12_ receptor mediates migration when activated by its ligands, ATP and ADP. Among brain cells, P2Y_12_ is primarily expressed in microglia, but not astrocytes or neurons (B. Barres, unpublished data, personal communication) [7], [8], [34], although it has also been described on oligodendrocytes [35]. In the peripheral circulation, it is largely found on platelets [8]. The P2Y_12_ receptor has been shown to induce microglial process extension and migration toward sources of ATP or neuron injury *in vitro* and *in vivo* [7], [10] as well is in human microglia [9]. P2Y_12_ has also been shown to be a key regulator in the induction of neuropathic pain following peripheral nerve injury and a regulator of microglial engulfment of injured myelinated axons [16], [36]. Our findings show that microglial P2Y_12_ provides a critical component in the pathway toward neurotoxicity following global cerebral ischemia, and extend the role of microglia in global cerebral ischemia. We show that deficiency of P2Y_12_ on microglia reduce death of neurons due to OGD, and this is accompanied by reduction of microglial migration in response to OGD-injured neurons and prevention of the formation of microglial clusters *in vitro*. These findings were further validated at the *in vivo* level, as mice deficient in P2Y_12_ suffered less ischemic injury to hippocampal neurons and decreased microglial activation.

Whether inhibiting P2Y_12_ would be beneficial in other neurological conditions, such as multiple sclerosis, Alzheimer’s or Parkinson’s disease, all of which are characterized by substantial pro-inflammatory responses, including microglial activation and ATP release, remains to be seen. There have been several reports of P2X_7_ receptor inhibition reducing disease progress in various models of chronic neurodegenerative disease [37], [38], [39], [40]. It should be noted that one key difference between microglia in acute ischemia versus chronic neurodegenerative conditions is that in acute brain injury, microglia undergo rapid transformation between their resting state to that of an activated state [41]. Since P2Y_12_ is expressed at the microglial membrane only in the cell’s resting state but internalized upon activation [7], it is possible that blockade of P2Y_12_ may only provide benefit acutely. Alternatively, if the internalization of the receptor, which is thought to occur upon prolonged exposure to ATP or ADP [1], [42], is required for downstream activation [43], then blocking or preventing its internalization may slow the progression of chronic neurodegenerative diseases. These are clearly areas for further exploration.

Since P2Y_12_ is also the target of the widely used antiplatelet drug, clopidogrel, we also showed that pharmacological inhibition was similarly protective and anti-inflammatory against global cerebral ischemia, a condition frequently seen following cardiac arrest. An obvious clinical utility of clopidogrel is that is commonly prescribed to patients with cardiovascular disease [44], and these patients are also at high risk of developing cardiac arrest. Thus, our study would suggest that these patients may have an additional benefit of prophylactic neuroprotection. We also show this neuroprotective effect when clopidogrel was administered orally, which was necessary not only out of translational relevance, but also because it is a pro-drug, requiring first pass metabolism through the liver to an active form [45]. Thus, clopidogrel cannot be studied in its current form *in vitro* or through parenteral administration. We chose to use the global cerebral ischemia model in this initial study, because we previously reported that this model does not lead to disruption of the blood brain barrier or allow significant entry of circulating blood cells [18]. GCI generally results in delayed neuron death of vulnerable populations of neurons, such as those in the CA1 region, as opposed to that which occurs in a focal infarct. Thus, we could avoid potential confounds such as any effect of clopidogrel on peripheral immune cells which might have infiltrated the ischemic brain. However, it is still possible that the effect of clopidogrel seen on brain microglia could be due to its direct effect on platelets, since we could not directly study this drug on microglia. Since clopidogrel is also a widely prescribed anti-platelet drug in patients at risk for ischemic stroke [46], future studies examining whether similar neuroprotection might be observed in an experimental model of focal cerebral ischemia should most certainly be pursued.

## Supporting Information

Figure S1 **Western blots of BV2 cells transfected with siRNA against P2Y_12_ (P2Y_12_ siRNA) or control siRNA (con siRNA) show approximately 50% decrease in protein.** Western blots of brain extracts from P2Y_12_+/− versus wildtype (WT) mice show approximately 75% decrease in protein. β-actin is shown as a housekeeping protein. **b:** Representative carbon black perfused brains to demonstrate scoring of posterior communicating arteries (PComm). Arrows point to the PComm. An animal with 2 PComms is given a score of 2, with only 1, a score of 1, and no PComms a score of 0.
(TIF)
