# Supplementary figures and images for: Microglial P2Y12 Deficiency/Inhibition Protects against Brain Ischemia
Source: PLoS One. 2013 Aug 5;8(8):e70927. doi: 10.1371/journal.pone.0070927 (PMC3733797; doi:10.1371/journal.pone.0070927)

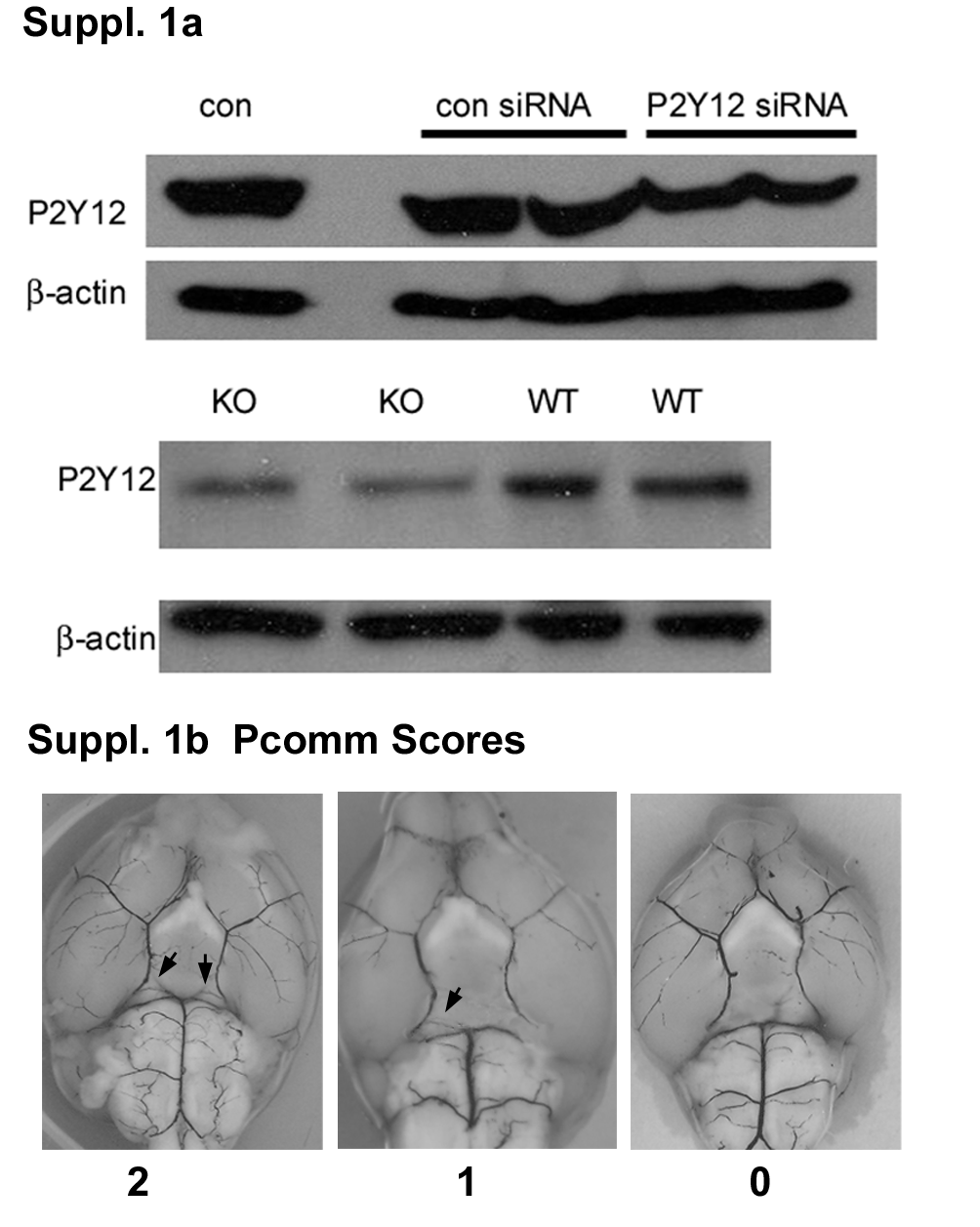

Supplement: Figure S1 — Western blots of BV2 cells transfected with siRNA against P2Y12 (P2Y12 siRNA) or control siRNA (con siRNA) show approximately 50% decrease in protein. Western blots of brain extracts from P2Y12+/− versus wildtype (WT) mice show approximately 75% decrease in protein. β-actin is shown as a housekeeping protein. b: Representative carbon black perfused brains to demonstrate scoring of posterior communicating arteries (PComm). Arrows point to the PComm. An animal with 2 PComms is given a score of 2, with only 1, a score of 1, and no PComms a score of 0. (TIF) [file pone.0070927.s001.tif]
